# Biomaterials for Protein Delivery: Opportunities and Challenges to Clinical Translation

**DOI:** 10.3390/mi15040533

**Published:** 2024-04-15

**Authors:** Amogh Gorantla, Jacques T. V. E. Hall, Anneliese Troidle, Jelena M. Janjic

**Affiliations:** 1 Department of Engineering, Wake Forest University, Winston-Salem, NC 27109, USA; goraa21@wfu.edu; 2 Department of Chemistry, Duke University, Durham, NC 27708, USA; jacques.hall@duke.edu; 3 School of Pharmacy, Duquesne University, Pittsburgh, PA 15282, USA; troidlea@duq.edu

**Keywords:** biomaterials, protein therapeutics, clinical translation

## Abstract

The development of biomaterials for protein delivery is an emerging field that spans materials science, bioengineering, and medicine. In this review, we highlight the immense potential of protein-delivering biomaterials as therapeutic options and discuss the multifaceted challenges inherent to the field. We address current advancements and approaches in protein delivery that leverage stimuli-responsive materials, harness advanced fabrication techniques like 3D printing, and integrate nanotechnologies for greater targeting and improved stability, efficacy, and tolerability profiles. We also discuss the demand for highly complex delivery systems to maintain structural integrity and functionality of the protein payload. Finally, we discuss barriers to clinical translation, such as biocompatibility, immunogenicity, achieving reliable controlled release, efficient and targeted delivery, stability issues, scalability of production, and navigating the regulatory landscape for such materials. Overall, this review summarizes insights from a survey of the current literature and sheds light on the interplay between innovation and the practical implementation of biomaterials for protein delivery.

## 1. Introduction

Incorporating biomaterials in protein delivery represents innovative strategies at the forefront of therapeutic interventions, yet still encounters several limitations and challenges toward clinical translation. Protein molecules are vital to nearly every biological process and are desirable in a clinical landscape as they can be produced in vitro on a massive scale while being minimal in cost, thanks to advancements in recombinant DNA technologies, cell culture, and biological separation. Thus, proteins have emerged as promising agents in the treatment of different human diseases [1]. For example, protein therapeutics that incorporate components such as enzymes, growth factors, and cytokines can be key in the treatment of conditions such as cancer and autoimmune, inflammatory, metabolic, infectious, and genetic disorders, as they have a high target specificity and a low toxicity in comparison to small-molecule drugs [2,3,4]. However, challenges to in vivo protein delivery stem from complexities in protein delivery mechanisms, protein therapeutics’ short half-lives, and their fragile structures [1,5]. Furthermore, the monitoring of therapeutic protein activity is highly susceptible to challenges such as interactions with other biomolecules and enzymatic degradation. Currently, though, there have been more than 200 protein therapies that have overcome such hurdles and have been approved by the FDA for wide-ranging therapeutic applications, with many more on the path toward such an approval and implementation [6]. These therapies depend on the ability of a drug delivery system to deliver proteins efficiently and precisely to the targeted site within the body [7,8] by navigating the complex biological pathways of the human body and selectively targeting diseased tissues while sparing healthy ones [9,10,11,12]. This task demands precision engineering and an intimate knowledge of physiological interactions, making it a focal point in current research and developments in the field. Among these interactions, structural instability caused by unfolding, misfolding, covalent/non-covalent modifications, and aggregation can degrade drug efficacy and greatly overshadow the bright features of these protein-based drugs [13]. The misfolded protein may turn into amorphous clots or aggregates with regular structures, each of which can lead to severe complications [14,15]. While great bounds are being made in the field, major gaps in progress still exist. Adenosine protein delivery systems are quickly referenced in such discussions because of their stability and targeted delivery profiles. Adenosine enhances the dilation of blood vessels throughout the body in the treatment of myocardial infarction, though vasodilation is only necessary within the heart—indicating a lack of efficient targeting [16]. Although adenosine has displayed possible medical benefits in individuals with central nervous system diseases, the bloodstream’s rapid metabolism as well as the inaction of multiple adenosine receptors in the body have made protein delivery systems difficult to use for adenosine delivery [17,18]. In both cases, protein delivery systems must adequately protect their payload and ensure a proper delivery [18]. Insulin delivery faces similar challenges, as the recurring injections which patients with diabetes must endure frequently inconvenience patients and cause them pain [19,20]. Oral protein delivery has also been proposed as an alternative route, especially given the significant progress in creating functional insulin-loaded nanoparticles and (more generally in the field of protein delivery) advancements in primary release mechanisms including diffusion, degradation of the biomaterial platform, swelling, and stimuli-triggered release (e.g., photorelease), among others [19,21,22,23,24,25]. However, we have found no oral insulin delivery systems presently on the market, as these advancements have not yielded a system which can pass clinical trials due to unclear toxicity, low oral bioavailability, and interference from nanoprotein interactions [19,21,22,26]. Thus, parenteral routes (i.e., subcutaneous, intravenous, and transdermal) have been the primary options for therapeutic protein delivery. Parenteral routes present with significant disadvantages over oral delivery, such as pain at the point of administration, potentially increased systemic side effects, risks of infection, reduced patient acceptance and adherence, and high costs [24,25,27]. Oral delivery could circumvent such pitfalls for protein therapeutics, though there are many obstacles which must be overcome, including protein stability, molecular size, and challenges in passing through biological barriers [28]. The gastrointestinal (GI) tract is an incredibly hostile environment with a high concentration of proteolytic enzymes that quickly degrade both nutritional and therapeutic proteins [29,30]. Furthermore, the intestinal wall represents a selectively permeable barrier that prevents larger macromolecules, such as proteins, from being absorbed [31,32]. To overcome these barriers and challenges, biomaterials emerged as an approach to therapeutic protein delivery [7,13,33]. A biomaterial is a natural or synthetic material used in medical applications for the purpose of enhancement, support, or replacement of damaged tissue or biological function [34]. Both synthetic and natural biomaterials can be used for protein delivery systems. Natural biomaterials, including chitosan, alginate, hyaluronic acid, collagen, keratin, elastin, genipin, silica, polysaccharides, polyphenols, and silk, are mostly non-toxic to humans due to their similarities with preexisting bodily molecules, cell-to-cell interactions, and structural support [35,36,37,38,39,40,41]. Therefore, these materials are often bioactive, providing adhesion regions for the surrounding cells and often degrading locally via a form of controlled release [42]. Although natural biomaterials outcompete synthetic ones in certain cases, synthetic biomaterials remove the mechanical hinderances of a natural material while also providing significant opportunities for specificity and tunability of the materials’ release. However, biomaterials are foreign bodies that sometimes result in adverse immune responses—a factor which can critically impact patients’ quality of life and impede progress toward effective treatment. These responses can present as inflammation, tissue degradation, a delayed healing process, and even immense pain, due to the immune system’s rejection of the biomaterial [42]. Given the burgeoning nature of this field, this review aims to explore and examine the impact of biomaterial integration in protein delivery systems by navigating the array of challenges and innovations on the path to clinical translation. We hope that this paper will serve as a beneficial resource, aiding researchers and clinicians alike in actualizing the clinical opportunities ahead.

## 2. Opportunities and Challenges

### 2.1. Viable Biomaterial Platforms for Therapeutic Protein Delivery

**Figure 1:**
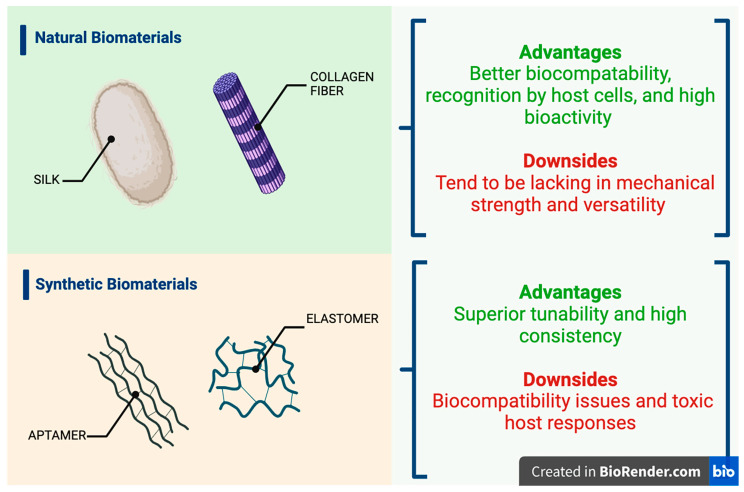
Various biomaterial examples, both natural and synthetic, which pertain to protein therapeutic delivery applications: advantages and downsides of natural biomaterials when attempting to formulate biomedical devices which utilize these for protein delivery. These materials can be fabricated into various drug delivery vehicles such as hydrogels, scaffolds, films, nanoparticles, and gels (**top**); advantages and downsides of synthetic biomaterials when attempting to formulate biomedical devices which utilize these for protein delivery—under research for drug delivery applications (**bottom**) (Created with BioRender.com).

From the late 1960s, researchers have investigated both natural and synthetic materials as drug carriers—focusing greatly on polymers. These biodegradable systems have been engineered specifically to prolong drug release and reduce the need for frequent and repeated administration, which is a leading cause of low patient adherence [43]. However, synthetic polymers can be challenging to control because of their structure and molecular weight, factors which directly correlate to unfavorable biodistribution and pharmacokinetics [44,45]. These synthetic biomaterials can also be incredibly expensive, difficult to scale for mass production, and present a myriad of regulatory challenges (Figure 1A) [46,47]. As a result of these drawbacks, the exploration of natural biomaterials such as lipids, polysaccharides, nucleic acids, and proteins as options for viable protein therapeutic delivery emerged [46,48]. Collagen, keratin, gelatin, and silk fibroin are all protein-based natural biomaterials that are utilized for a variety of biomedical applications such as films, hydrogels, and microparticles. Keratin is an example of a protein that is employed in the formulation of inks for a variety of biomedical applications with high biocompatibility, biodegradability, availability, affordability, and tunability (Figure 1B) [42,49,50,51,52]. Furthermore, collagen is highly suitable for drug delivery platforms; however, it can be challenging to process via heat as a high temperature is required during material processing [42,53,54]. By using these biomaterials and others, researchers have been able to enhance the functions of many protein delivery systems. A condensed discussion of natural and synthetic carriers for proteins can be found in Table 1, with results based on their chemical structure.

#### 2.1.1. Hydrogels

Hydrogels, for example, can be defined as a network of 3D hydrophilic polymers that can absorb a large quantity of water. These biomaterial devices are highly tunable as they can be formulated into many different shapes, formats, and out of a variety of polymers [1,64]. Pioneering studies by Davis, Langer, and Folkman indicated such promise for protein delivery via hydrogels throughout the 1970s. Davis incorporated a polyacrylamide (PAA) hydrogel loaded with insulin for patients combatting diabetes and discovered that protein diffusivity was inversely proportional to the molecular weight, which was inversely logarithmically dependent on the polymer concentration [1,65]. Langer and Folkman showed a largely similar work to Davis’, which indicated many of the larger challenges in protein delivery such as rapid and abrupt release kinetics and the impact of harsh components for hydrogel crosslinking [1,66]. Currently, a variety of polymers are used in hydrogels for protein therapeutic delivery, such as the following: poly (ethylene glycol) (PEG), 2-hydroxyethyl methacrylate, poly (vinyl alcohol) (PVA), methacrylic acid (MAA), and N-isopropyl acrylamide [42,56]. Additionally, there are many types of hydrogels that have varying degrees of applicability for protein delivery: (1) Traditional hydrogels retain proteins physically within their matrix without specific binding sites, leading to a simple design but often resulting in poor protein sequestration and significant burst release. However, traditional hydrogels do not always follow burst release kinetics. For example, in a 2015 study, Yu et al. reported a formulation of a hyaluronic acid and dextran-based hydrogel loaded with bevacizumab to be administered via intravitreal injection for the treatment of eye disease in a rabbit model [67]. In their study, the six-month retention of bevacizumab within the eye was observed and was 10^7^ higher in concentration than that in the bolus injection group [67]. While other hydrogels may have higher-affinity binding sites which exhibit minimal opportunities for burst release of their therapeutic, this hydrogel was formulated by mixing the two polymers and therapeutic protein [67]. In this instance, the properties of the two polymers contributed to the controlled release of the therapeutic, as hyaluronic acid is known for its slow degradation in the eye [67]. By optimizing hydrogel materials, one can finely tune traditional hydrogel for the controlled release of their desired therapeutic. (2) Heparin-based hydrogels utilize heparins as protein-binding sites, with adjustable binding affinities. They show low burst release but have safety concerns due to animal tissue derivation and a low specificity. Heparin has previously shown affinity to bind growth factors, and this characteristic is often utilized for embedding specific growth factors into a heparin-based hydrogel for improved growth factor release [68]. In a 2011 report, heparin-based hydrogels were specifically used as a matrix to encapsulate primary hepatocytes when incorporated with the hepatocyte growth factor (HGF) [68]. In the aforementioned article, cell viability was significantly higher in the heparin-based hydrogels than in the PEG-hydrogels used as a control in the experiment [68]. In this instance, specific binding sites allowed for an improved therapeutic delivery of proteins when compared with non-specific and simple designs previously described for traditional hydrogels. (3) Peptide-based hydrogels feature peptides as the binding sites for proteins, with tunable binding affinities. They offer reduced burst release and a high biocompatibility but suffer from a low binding affinity and a high peptide-to-protein ratio [69]. In their 2021 article, Gallo et al. reported a peptide-based hydrogel for the delivery of Doxorubicin, a commonly prescribed antitumoral drug, and showed that, when encapsulated in either Fmoc-FF (9-fluorenylmethyloxycarbonyl-diphenylalanine) alone or a modified Fmoc-FF, the cell viability of cells from an aggressive triple-negative breast cancer (TNCB) cell line was reduced by nearly 50% when compared to the empty hydrogel used as a control measure in the experiment [70]. Peptide-based hydrogels have been investigated to encapsulate nanodrugs for the targeted delivery and elimination of systemic toxicity. (4) Aptamer-based hydrogels employ aptamers as the protein-binding sites, which allow for high affinity and specificity [71]. They excel in low burst release, high biocompatibility, and controlled release but are limited by the availability of aptamers. These aptamers can fold into the 3D conformations of the proteins’ tertiary structure for enhanced binding specificity and have been commonly employed for molecular detection, cell detection, and targeted therapeutics [72]. DNA-crosslinked hydrogels have been reported for targeted drug delivery through the grafting of azobenzene-incorporated DNA into a hydrogel for the controlled release of anti-cancer drugs when exposed to high temperatures and targeted to tumor tissues [73]. Among these listed hydrogel types, with different ligands for protein recognition and physical immobilization, aptamer-based hydrogels stand out for their high affinity, specificity, and ability to control the spatiotemporal release of proteins, making them a promising option for precise and efficient protein delivery via diffusion coupled with a binding reaction. It is important to note that the fabrication of the given hydrogels can be risky, impacting the therapeutics’ stability. Safeguards during the processing and storage of the platform are vital to avoiding issues such as aggregation and denaturation [1,74].

#### 2.1.2. Scaffold Systems

**Figure 2:**
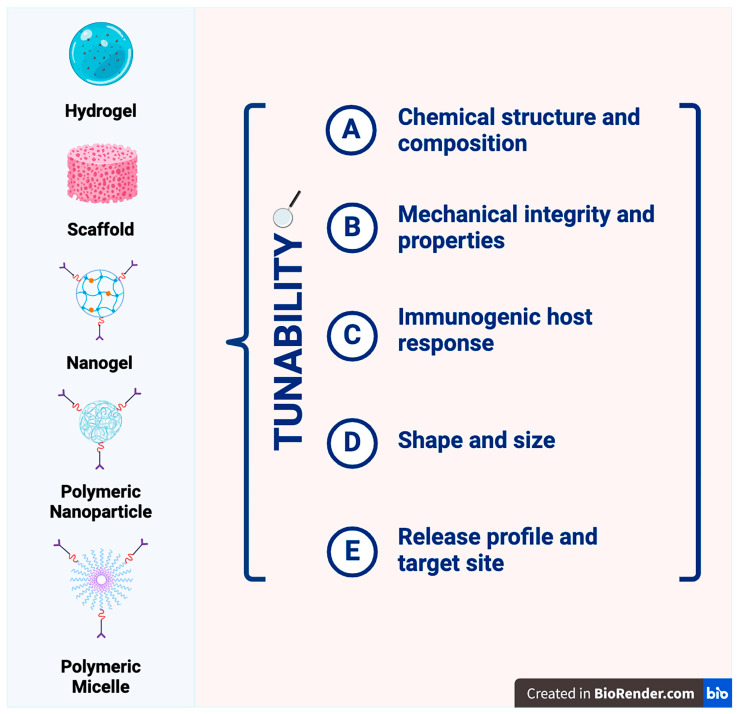
Different examples of viable biomedical devices for protein delivery. Analysis of tunable factors for the different devices, emphasizing how these platforms are potential solutions to circumvent issues with standards of care (oral and parenteral routes).

Like hydrogels, scaffold systems are a class of medical devices that can be fabricated from different biomaterials. These can be engineered to mimic different tissues, provide cues to immune cells, and prompt desirable effects such as tissue repair and regeneration. However, the variety of synthetic and non-synthetic materials that can be used in such scaffolds can result in varied protein payload stability [75]. Scaffolds are incredibly versatile, as they can incorporate cytokines and growth factors to modulate immune responses and are retrievable post implant/injection, which makes monitoring in vitro release much easier (Figure 2) [42,76,77]. Scaffolds of both natural and synthetic biomaterial origin have been implemented in therapeutic measures for protein delivery across various fields of medicine, including the management of orthopedic diseases such as osteoporosis and the regeneration of peripheral nerves from nerve injury, as well as many other applications. The fabrication of nerve guidance channels (NGC) out of poly(2-hydroxylethly-methacrylate-co-methyl-methacrylate) (P(HEMA-co-MMA)) with the nerve growth factor (NGF) incorporated into the inner layer of the NGC allowed for the controlled release of NGF and other factors necessary for tissue regeneration [78]. Scaffolds will continue to be commonly used in biomaterial research and therapeutics’ development, especially in research pertaining to tissue regeneration and tissue engineering.

#### 2.1.3. Nanogels

Nanosystems, including nanogels, polymeric nanoparticles, and polymeric micelles, are emerging biomaterials for therapeutic protein delivery. Nanogels are characterized as polymer particles on the nanometer scale with 3D networks of polymer chains formed via crosslinking that can swell in an aqueous solvent [79]. In the swollen state, the nanogel can sufficiently encapsulate materials like proteins via van der Waals forces and electrostatic interactions between the payload and the nanogel polymers [79]. Nanogel polymer crosslinking takes place either via chemical or physical methods. Nanogel crosslinking includes irreversible linking via covalent bonds, reversible hydrogen bonds, hydrophobic interactions, and other non-covalent molecular interactions [80]. Nanogels function very similarly to hydrogels, just at the nanoscale, which allows for the more efficient encapsulation and carrying of biologically active molecules, thereby increasing the stability of their protein payload [81]. Their extremely small size allows for longer circulation and avoidance of rapid clearances as well as enhanced permeability toward the epithelium, a particularly advantageous characteristic when developing therapies targeting cancers [81]. A study performed in 2008 found that, when a nanogel containing trastuzumab, an FDA-approved drug for treating HER2-positive breast cancer, was administered to a cell line containing HER2-overexpressing tumor cells, significant toxicity was observed, but, when administered to a cell line with normal HER2 expression, no toxic effect was observed [82]. Furthermore, certain nanogels can be designed to not elicit an immunological response from the host [81]. With this example and others, one can envision the promise of nanogels as versatile vehicles for therapeutic protein delivery.

#### 2.1.4. Polymeric Nanoparticles

Polymeric nanoparticles include particles smaller than one micrometer in diameter that contain bioactive materials and can be loaded within or onto a polymeric core [83]. Polymeric nanoparticles can be classified as either a nanocapsule or a nanosphere, both of which are able to contain drug or protein compounds for targeted delivery and follow first-order and zero-order release kinetics, respectively [83]. Typically, polymeric nanoparticles exhibit high structural integrity and storage stability, as well as controlled release mechanisms [84]. These nanoparticles function very similarly to nanogels and differ only in that they are not embedded within a hydrogel matrix. Closely related to polymeric nanoparticles are liposomes, which have improved biocompatibility due to their structure closely resembling that of biological membranes, thought to function similarly to nanoparticles. Further research has delved into the combination of liposomes and polymeric nanoparticles, resulting in the development of lipid–polymer hybrid nanoparticles (LPNs) which combine the advantages of both systems while making up for the limitations of each system [84]. Polymeric micelles closely resemble polymeric nanoparticles and liposomes in that they have a nanoscale core surrounded by an amphiphilic block of copolymers [85]. They too have been widely used for drug delivery and have shown their ability to attenuate systemic toxicity, enhance targeted delivery, and improve efficacy [85].

It is important to note the differences in nanogels and the affinity of hydrogel platforms to that of traditional hydrogels, which include the following: (1) nanogels differ primarily in size, within the scale of nanometers, enabling precise delivery better than other gel platforms; (2) affinity hydrogels contain a trigger capability and, thus, have specific indicators that enable their release and activity in specific scenarios [1,81]. A condensed discussion of the benefits and limitations of each previously discussed biomaterial platform used for protein delivery can be found in Table 2.

### 2.2. Efficient and Targeted Delivery: The Core Challenge

**Figure 3:**
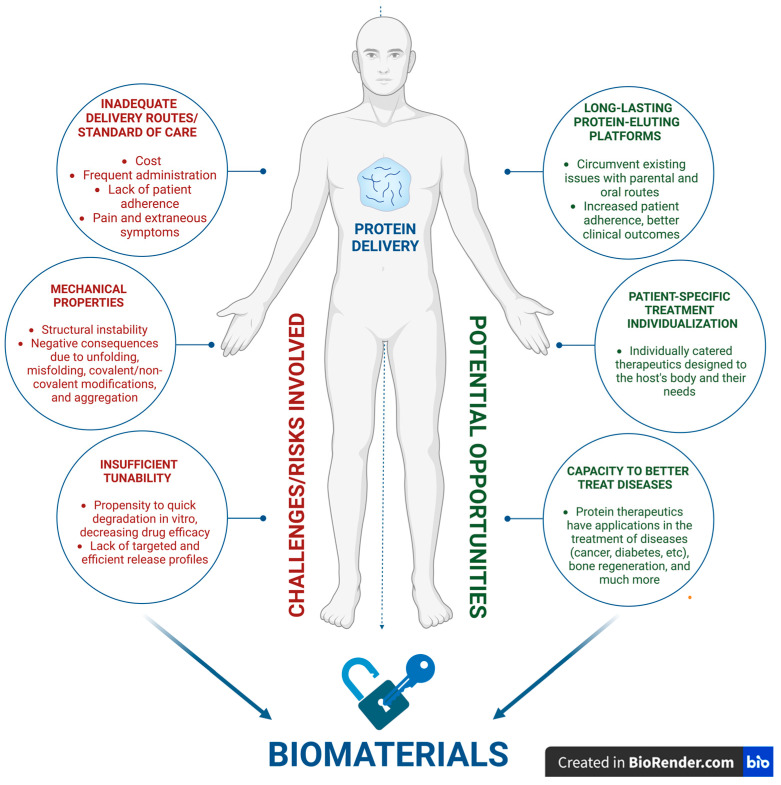
Modern challenges and opportunities associated with protein delivery for clinical applications of biomaterials, and a variety of biomedical devices fabricated from these could help mitigate said challenges, assisting in translation and furthering positive patient-oriented outcomes.

Though protein therapies are highly favored by researchers, they still face a hindering obstacle on the road toward clinical translation: efficient and targeted delivery mechanics. In nearly all cases, such therapies succeed because they both protect the protein’s structure from degradation in order to ensure the protein’s efficacy and simultaneously interact specifically with the corresponding target site in the body [2,24,125]. Although protein-based therapeutics show a high specificity at much lower concentrations than small-molecule drugs, integrating biomaterials into these therapeutic systems can help in overcoming difficulties through efficient and targeted delivery [8,126]. Biomaterials would allow for effective treatment without risking a high variability in drug circulation and concentration. In many cases, the application of protein therapeutics via traditional routes results in issues such as significant non-specific tissue–organ interactions, poor stability and solubility, adverse effects, and longevity issues, which, in turn, potentially lead to drug waste and extraneous costs for both patients and clinicians (Figure 3) [127]. Biomaterials would assist in such problems by acting as facilitators of sustained release where the drug would require less frequent administration to minimize issues with patient adherence and allow for a greater degree of control on drug release kinetics and circulation throughout the body [128]. For example, utilizing elastin-like polymers (ELP) like Poly(VPAVG) for bone regeneration demonstrated great success in terms of encapsulation efficiency, biological activity, and release profile of the therapeutics [127,129,130]. Biomaterials such as ELP could significantly assist in improving protein therapeutic delivery by allowing for the inclusion of factors such as self-assembly triggers and enzymatic degradation sequences, which, overall, further the targeting potential within platforms such as nanocarriers, aggregates, micelles, and hydrogels [127].

### 2.3. Addressing Immunogenicity and Biocompatibility: Safety, Toxicity, and Tolerability 

The interaction between protein molecules and the body’s immune system presents a significant challenge in developing protein-based therapies. The introduction of foreign proteins can trigger immune responses, potentially leading to adverse effects and diminishing a therapy’s effectiveness. Furthermore, the stability of protein molecules is of paramount importance, and these molecules are prone to degradation when exposed to the body’s biochemical environment [2]. There is also immense difficulty in predicting the immunogenic response to protein therapeutics, resulting in potentially life-threatening responses, such as anaphylaxis, and reduced efficacy overall, with the most common response being a high-affinity, anti-therapeutic, antibody response [131]. Because most therapeutic proteins have a potential for such issues, it is essential to quantify and analyze these issues by assessing molecule-specific immunogenicity using methods like anti-drug antibody detection [132]. Nevertheless, there are a multitude of issues with preclinical testing methods in animal models, as these findings are often difficult to translate to a human clinical setting. For this reason, different computationally driven models can be used as a viable alternative [131,132]. Biomaterials are being developed to both stabilize proteins and minimize immunogenic responses. Regarding the decision between natural or synthetic biomaterials, natural biomaterials offer several advantages, such as high bioactivity and recognition by host cells, and thus reduce immunogenic responses in comparison to synthetic alternatives [46,125]. When analyzing many traditional delivery pathways (intravenous, subcutaneous, etc.), immunogenicity, formulation, and anti-drug antibody response issues due to frequent and repeated administration are also factors that can accumulate in negative and even toxic ways [133,134,135]. These issues, along with negative cumulative effects caused by an abrupt burst release of therapeutics, highlight the importance of biomaterial-assisted drug delivery systems like those mentioned previously in minimizing these problems [29,77,136]. Biomaterials can facilitate protein delivery by releasing proteins at the right time and location within the body, ensuring optimal therapeutic effects through more controllable and targeted release profiles. Biomaterials as a solution for protein delivery will allow the bright features of protein-based drugs to outshine the negative consequences resulting from structural instability due to unfolding, misfolding, or covalent/non-covalent modifications, as well as aggregation, thereby reducing drug efficacy [125]. 

### 2.4. Stability Issues

Stability issues are crucial to consider when either developing or applying biomaterials to protein delivery. Proteins are complex molecules that are sensitive to environmental factors like temperature, pH, and proteolytic enzymes, which can lead to denaturation. Once denatured, the proteins lose their tertiary structure and could become therapeutically inactive, while still able to cause an adverse immune response [16,137]. Therefore, delivery systems that use biomaterials that can maintain a protein’s structure and function during its delivery can enhance stability, although considering which materials and type of delivery system to use is essential because delivery vehicles degrade for both material and environmental reasons. Certain nanocarriers with a low crystallinity, including those built with inert materials, and with a larger particle size are more easily degraded. When exposed to a lower pH, the calcium ions interact with a calcium-containing nanocarrier, and, when then exposed to the phosphate ions in bodily fluids, they are more easily degraded [138,139,140,141,142,143]. These nanocarrier stability considerations must also be discussed because the biomaterials in a nanocarrier may unfavorably interact with the protein, causing aggregation, adsorption, or conformational changes which can change the therapeutic efficiency and/or safety profile [144,145,146]. Controlled release is also essential in protein delivery systems, meaning that the release mechanism must ensure that therapeutic protein delivery occurs in the right place, quantity, and time. However, controlling the release often means changing the structure of the delivery systems such that the proteins are more tightly encapsulated or have stronger biomaterial (e.g., nanogel) matrix–protein interactions, which, in turn, could increase the risk of protein denaturation or aggregation [147]. The balance between controlled release and maintaining the stability of the protein payload presents a challenge for both the design and manufacturing of protein delivery systems. It is of utmost importance that research is carried out to establish a methodology that allows for the production of highly environmentally stable protein-based therapeutics for the optimal and efficient delivery of therapeutic agents in a controlled manner.

### 2.5. Production Scale-Up and Navigating Regulatory Challenges 

Translating the success of biomaterial-based delivery systems from laboratory prototypes to widely available treatments poses substantial challenges. Scaling production involves overcoming logistical, economic, and technical barriers to ensure the consistent quality and affordability of these advanced therapies [148]. The evolution of production methods, from small-scale laboratory settings to large-scale manufacturing, requires a thorough understanding of the processes involved and the ability to adapt these processes to meet the demands of mass production [31,149]. In a 2020 report on the manufacturing of nanoparticles for pharmaceutical applications, a scale ranging from 100 g to 12,000 g was compared in terms of manufacturing methods and reproducibility throughout the wide range of production [150]. In this study, high-pressure microfluidization was utilized to convert the coarse emulsion into a fine nanoemulsion before the extraction of the organic solvents to leave researchers with hardened nanoparticles [150]. When comparing the size of the nanoparticles across the batches, only a 10% variability in size was observed, even though the change in size of the batch was more than 100 times greater [150].

The manufacturing of biomaterials for protein delivery presents a multitude of technical challenges. For example, when manufacturing hydrogels and similar biomaterials, sterilization methods, the evaluation of shelf and long-term stability of the product, and route of delivery must be taken into consideration [151]. Traditional sterilization techniques that include the use of high temperatures may not be a plausible option, especially in the case of thermoresponsive hydrogels or if they contain proteins which are sensitive to high temperatures or large temperature fluctuations. Filtration is commonly used but, depending on the filter pore size required for the sterility assessments and viscosity of the biomaterial, the integrity of the therapeutic agent may be compromised. Chemical methods of sterilization are also potential avenues to investigate, but one must ensure that exposure to the sterilizing liquid will not lead to a reaction in the biomaterial being sterilized to avoid altering the function of the biomaterial. Shelf life and long-term stability are also important to monitor because, in large-scale production, the biomaterials are purchased in bulk from the supplier and stored as noted. Understanding the timeline of when a biomaterial will begin to no longer function as intended is important to prevent adverse reactions in a clinical setting. In the case of hydrogels, crosslinking oftentimes has a limit in terms of their overall stability, and they will eventually destabilize when stored in their liquid form for too long [151]. Finally, the physical characteristics of the biomaterial being produced will dictate the route of administration to the patient. If viscosity is too high, parenteral administration is likely unfeasible. If the viscosity is too low, topical administration is not likely to be an option. These are all important factors to consider during the formulation and manufacturing process of therapeutic protein delivery agents.

Additionally, the regulatory landscape for novel biomaterials presents its complexities [152]. These materials require rigorous testing and compliance with evolving health regulations, a process which is both time-consuming and resource-intensive. The regulatory aspect is critical in ensuring the safety and efficacy of biomaterials for protein delivery, requiring a collaborative effort between scientists, engineers, and regulatory bodies. Firstly, they engage in rigorous preclinical testing to ensure safety and efficacy, complying with standards set by regulatory bodies like the FDA and EMA. Secondly, there is a focus on transparency and ethical considerations, especially regarding the sourcing and sustainability of the biomaterials. Finally, collaboration with regulatory authorities during the development phase is crucial, ensuring that innovations align with evolving regulatory frameworks and guidelines. This approach helps in addressing the complex regulatory landscape governing the use of biomaterials in protein delivery systems.

### 2.6. Advancements in Biomaterial Fabrication Technologies 

Advancements in fabrication technologies, particularly 3D printing, are reshaping the prospects of biomaterials in protein delivery. These technologies enable the design and creation of highly complex structures tailored to specific therapeutic needs and individual patient profiles. Three-dimensional bioprinting enables scientists to build biomaterial scaffolds with control over the minute details of their size, shape, and features. This allows for the creation of scaffolds that mimic the extracellular matrix to improve cell–material interactions and promote tissue regeneration [42,153,154,155,156]. The incorporation of proteins and/or growth factors into these scaffolds enables scientists to utilize them as localized and sustained drug delivery systems, which is of particular importance for applications in which controlled release is essential, like wound healing, bone regeneration, and the treatment of chronic diseases [157,158,159]. In a 2014 study published in Nature Communications, 3D decellularized extracellular matrices (ECM) were printed using novel bioink to serve as scaffolding for adipose, cartilage, and heart tissues [160]. Tissue from donor hearts, adipose, and cartilage was harvested, decellularized, and solubilized to leave the remaining ECM in gel form [160]. This gel was then printed as dECM bioink, laden with cells, into a polymer framework [160]. Upon gelation, the 3D-printed cellular structure was successfully used for in vitro disease modeling, drug screening, and tissue engineering applications [160]. The inclusion of cell- and tissue-specific proteins allowed for the 3D-printed cell structure to continue to function for the in vivo studies being performed. Three-dimensional bioprinting has continued to be optimized and used for even more therapeutic applications since its advent and will continue as research continues to progress.

Electrospinning is another fabrication technique by which ultrafine fibers are derived from polymer solutions. Electrospun fibers form non-woven mats with high surface area-to-volume ratios, which are particularly important in protein delivery, as the mats’ high loading capacity and protective encapsulation of the protein make them favorable delivery systems [161,162]. The controlled release of proteins is accomplished using electrospun fibers by manipulating their composition, alignment, and degradation rate [133].

The field has also gradually began shifting toward “smart” delivery systems that respond to an array of stimuli such as pH, temperature, oxidative conditions, the presence of different biomolecules, and host responses, and have unique properties and incredible tunability [46,163]. By integrating smart materials into one of these delivery systems, the systems gain the ability to respond to external stimuli like temperature or pH, enabling an even more precise control over the release of proteins and reducing side effects and, thereby, patient non-compliance [164]. For example, certain multilayered scaffolds and hydrogels can release different active molecules when the body indicates a certain healing phase or immune response; however, success with a polymer-based stimuli-responsive smart biomaterial is still a much more novel idea, as a far deeper understanding of the human body, diseases/stimuli, and interactions between human immune cells and biomaterials [42,165,166]. 

### 2.7. Nanotechnology, Hybrid Biomaterials, and Bioprinting—The Next Frontier

The field of protein delivery has a plethora of future frontiers that have arisen due to significant advancements in precision targeting technologies, specifically in the areas of nanotechnology, hybrid biomaterials, and bioprinting. In recent times, nanotechnology has been playing an even more important role, primarily through the use of nanoparticles as protein delivery systems [33,167,168]. These particles are designed to be miniscule, with a single particle being, in some cases, only a few nanometers large, their small size permitting easy penetration through a variety of biological membranes and barriers [169,170,171]. The particles themselves can be designed to decrease the degradation rate, increase the half-life, and moderate the release rate. Furthermore, accessory targeting ligands can be added to the surface of the particles, thereby guiding the particle to bind to a selective cell type, aiding in its precision-targeting capabilities [172,173,174,175]. Specifically, this precision-targeting ability has been utilized the most within cancer therapeutics, wherein precision in delivering cancer-killing agents is essential [164,175,176,177]. Hybrid biomaterials are another frontier in the field, combining the properties of multiple materials like metals, ceramics, or polymers to generate delivery vehicles with either a combination of those materials’ properties or with entirely new functionalities [178,179,180,181,182,183]. They are most often used to aid in precision targeting because of their controlled release; by using a variety of ingredients to engineer these vehicles, they can be engineered to respond to specific stimuli [184]. Bioprinting technology is the last of these three frontiers and can create three-dimensional scaffolds, which, in the context of protein delivery, can be loaded with proteins and/or growth factors [52]. Thus, these scaffolds can be designed so that they mimic the natural extracellular matrix, creating an environment which is advantageous for cell growth and tissue regeneration [185,186]. They can release proteins in a targeted manner by altering the composition and structure of the protein itself to enable the sustained and localized delivery of said protein [42,158]. These technologies represent significant opportunities for improving the targeted, controlled, and efficient delivery of therapeutic proteins, opening new pathways for the treatment of diseases and injuries via a new generation of protein delivery systems.

To reiterate and further elaborate upon our prior statements, examples of protein therapeutics that have reached the landmark of clinical translation include erythropoietin for chronic kidney disease and chemotherapy, insulin for the treatment of diabetes, interferons for multiple sclerosis and hepatitis B and C, and monoclonal antibodies such as trastuzumab for breast cancer and infliximab for rheumatoid arthritis and inflammatory bowel disease. In conjunction with positive innovations stemming from biomaterial-oriented delivery methods, novel protein therapeutics will undoubtedly be created and improved upon in the future [187].

## 3. Conclusions

Currently, many opportunities associated with protein delivery are yet to be fully grasped due to inherent challenges in delivering protein molecules, potential negative host responses through traditional and novel delivery routes, and complexities in formulating biomaterial-based solutions. These solutions, encompassing synthetic and natural options across various platforms like scaffolds, hydrogels, nanoparticles, and aggregates, present a high degree of variability in their formulation, material, and release profiles. In synthetic and natural polymers, each one exhibits unique properties and limitations. Synthetic biomaterials, like elastomers, aptamers, and other polymers, offer precise tunability and consistency and yet may pose biocompatibility and toxicity challenges. Natural polymers, such as collagen and silk, offer excellent biocompatibility and bioactivity, making them more suitable for applications such as the delivery of protein therapeutics, where the natural interaction with body tissues is crucial. However, some of their drawbacks include a general lack of mechanical strength and versatility when compared to synthetic polymers. Scaffolds and hydrogels, as examples of recent efforts in biomaterial development, stand out for their ability to mimic the natural extracellular matrix, thereby supporting cell growth and protein delivery. Hydrogels are advantageous for their high degree of tunability for factors such as porosity and hydration, which are essential for the controlled and sustained release of proteins. Nanosystems such as nanogels, polymeric nanoparticles, and polymeric micelles, distinguish themselves at the forefront of the field by leveraging their nanoscale precision, unique physicochemical properties, and capacity for fine-tuned delivery mechanisms to navigate biological environments effectively, offering a new dimension in drug delivery technologies. Controlled release is vital to ensure a targeted and efficient delivery to specific sites in the body, minimizing systemic side effects and immunogenic responses. Addressing stability issues, such as protein denaturation and aggregation, is critical. Nanocarriers, for instance, play a pivotal role in preserving the functional integrity of proteins, ensuring their stability and efficacy. Diseases for which protein delivery would prove greatly beneficial would be assisted significantly by these advanced delivery systems. Synthetic biomaterials like elastin- and aptamer-based materials are being explored for their specific binding capabilities and responsiveness to environmental stimuli. These properties are also crucial for targeted delivery and releasing therapeutics within the desired therapeutic range. However, it is evident that the path to clinical translation is far from being straightforward. The challenges include costs, safety, regulatory compliance, and scalability. Biomaterial devices are an essential alternative approach to overcoming issues associated with delivery routes (oral, subcutaneous, and intravenous) like patient adherence, pain, and frequent administration. Thankfully, the research in this field is exponentially blossoming, and novel advancements in fabrication technologies, particularly in bioinks and 3D printing, are driving the field forward. These technologies enable the creation of highly individualized delivery systems tailored to each patient’s needs and physiology, furthering the potential of personalized medicine. Ultimately, while biomaterials present a promising avenue for protein delivery, the journey toward fully harnessing their potential involves navigating a landscape riddled with scientific, clinical, and regulatory challenges. The future of this field lies in continued research and collaboration, focusing on overcoming these barriers and pushing the boundaries of what is currently possible in protein therapy.

## Figures and Tables

**Table 1:** Examples of characteristics for various commonly utilized synthetic and natural carriers.

**Natural Carriers**	**Results Based on** **Chemical Structure**	**Synthetic** **Carriers**	**Results Based on** **Chemical Structure**
Silk Fibroin	Prepared and processed to mitigate the loss of bioactivity Regulates structure and morphology of silk Stabilization due to binding interactions with its silk subunits Biocompatible Less inflammatory than other common biodegradable polymers Mechanically durable Controllable degradation rate via crystalline state during processing Regulates release profile of bioactive molecules Controlled degradation via surface digestion by proteases vs. bulk digestion [2,35,55]	Poly (ethylene glycol) (PEG)	Vast diversity in macromer chemistry and molecular weight, leading to finer tunability Excellent biocompatibility and non-toxicity Repeating ether units makes PEG ○ Hydrophilic and water-soluble ○ Relatively inert to many chemical reagents ○ Relatively elastic/flexible Stable under normal conditions and does not easily degrade PEGylation (attaching PEG units to a molecule) can lower immunogenicity of target molecule [42,56]
Cellulose	One of the most prevalent biomaterials on the planet Does not produce an inflammatory response Does not get absorbed in tissues because they are unable to manufacture cellulase Can be used to formulate controlled delivery mechanisms Can be derived from a microbial origin—better mechanical properties and biocompatibility Swelling capabilities, high biodegradability Unique encapsulating and binding properties Can change the solubility or gelling behavior of drugs Regulation of drug release [39]	Poly (vinyl alcohol) (PVA)	Repeating hydroxyl units make PVA ○ Water-soluble ○ Hydrophilic Satisfactory tensile strength, flexibility, and adhesiveness Biodegrades under most conditions to water and carbon dioxide, making it non-toxic Forms clear, glossy films with excellent barrier properties, though they are sensitive to strong acids and bases [42,57,58,59]
Heparin	Low molecular weight Can have a pronounced anticoagulative effect Through negative charge, it can stabilize proteins containing basic lysine and arginine residues through electrostatic interactions In certain cases, the high molecular weight can result in unwanted bleeding in certain applications Growth factor binding, and apoptotic and antiangiogenic effects [2]	Methacrylic acid (MAA),	Highly reactive due to the presence of both vinyl and carboxylic acid group Soluble in water and most organic solvents Used to introduce crosslinking sites into polymers, enhancing their mechanical strength, thermal stability, and resistance to solvents and chemicals Can be engineered to be non-toxic and non-immunogenic [56,60]
Hyaluronic Acid	Highly adaptable, biocompatible, biodegradable, nonimmunogenic, natural degradability Good absorption widely distributed throughout the body—found in a variety of human tissues such as eye vitreous humor, arthrodial cartilage, skin, and umbilical cord Specific binding affinity to CD44 receptor, a tumor-targeting ligand Can also serve as a protective shell for the nanocarriers for protein delivery [2]	N-isopropyl acrylamide (NIPAAm)	Physical and chemical stability Non-toxic degradation products Lower critical solution temperature allows for NIPAAm to alternate between soluble and insoluble stages with temperature changes Isopropyl group in NIPAAm provides hydrophobic characteristics while the acrylamide provides hydrophilic characteristics, creating a hydrophilic–hydrophobic balance, which can be adjusted to optimize the polymer’s solubility, swelling, and payload interactions Can be used to make polymers responsive to stimuli like pH changes [56,61]
Starch	Applications in nanotechnology, inexpensive, non-toxic, renewable, biodegradable Suitable for use with a variety of different materials in industrial application Nascent starch is unfeasible for sustained release due to gradual swelling and faster enzymatic decomposition Higher-enzymatic-degradation alternatives exist [55]	Polyacrylamide (PAA)	Generally biocompatible, non-toxic, and inert Presence of amide groups make PAA very hydrophilic, which facilitates the encapsulation of the protein Network structure of PAA can be altered to create gels with specific pore sizes, making size-based separation much easier to achieve [56,62]
Chitosan	Non-toxic, biocompatible, antibacterial, hemostatic, biodegradable Higher mechanical strength bears merit for hybrid biomaterials Mucoadhesive and intestinal epithelium-penetrating properties—beneficial for oral delivery pathways Soluble in acidic aqueous conditions Can be used to exploit antimicrobial properties [2]	2-hydroxyethyl methacrylate (HEMA)	Biocompatible, chemically stable, temperature-resistant, and hydrophilic Strong crosslinking ability, which protects and stabilizes the encapsulated protein Water content in Poly(HEMA) hydrogels can be adjusted such that gas and nutrient permeability is regulated HEMA can be fabricated in many ways (i.e., thermal or UV-polymerization) [56,63]

**Table 2:** Summary of different medical devices used in protein drug delivery systems.

Biomaterials	Advantages	Disadvantages
Traditional Hydrogels	Minimize adverse immune response risk due to their high biocompatibility [86] Exhibit controlled and sustained release to maintain therapeutic levels and reduce dosing frequency [87]	Can excessively swell or degrade in biological conditions, reducing structural integrity, which leads to the premature release of proteins [88]. Can lose effectiveness and stability when dosed with a high-protein concentration [89].
Affinity Hydrogels	Tissue or cell-specific, reducing off-target effects [86] Can be engineered to be stimuli-responsive to a variety of environmental factors [87]	Require precise control over their chemical properties so that they properly interact with their protein target [57] Binding can interfere with the protein’s active sites or induce conformational changes [90]
Scaffolds	Structures mimic the extracellular matrix, promoting cell attachment, proliferation, and differentiation [91] Can be designed for controlled release [92]	Risk of inflammatory response and/or rejection [93] Expensive fabrication [94]
Nanoparticles	Protect proteins from enzymatic degradation [95] Target-specific [96]	Exhibit toxicity or elicit an immune response upon accumulation [97] Expensive and difficult to manufacture [98]
Microparticles	Sustained and controlled release [99] Create a protective environment for the proteins [100]	Can aggregate and destabilize [101] Size can lead to difficulties in targeting smaller sites in the body [102]
Micelles	Increase the solubility of hydrophobic proteins in aqueous environments [95] Can specifically target one cell or tissue [103]	May face stability issues in the bloodstream [104] Limited loading capacity for proteins [105]
Aggregates	Protect therapeutic proteins from degradation and denaturation [106] Sustained release mechanism [107]	Can elicit immune responses [108] Difficulty controlling size and uniformity of aggregates [109]
Electrospun Fibers	High surface area-to-volume ratio [110] Can have controlled release [111]	Processing can denature protein [112] Inconsistent processing for non-uniform products [113]
Buccal/Sublingual Films	Rapid absorption of proteins through mucosal tissue [114] Bypass the GI tract and the first-pass metabolism in the liver [115]	Size restricts the amount of protein that can be loaded [116] Prolonged or repeated use can cause irritation to mucosal tissues [117]
Liposomes	Protect proteins from degradation by enzymes and harsh gastrointestinal conditions [118] Surface modifications can lead to improved target specificity [119]	Can be unstable and have premature leakage of the encapsulated proteins or fusion with other liposomes [120] Difficulties with inconsistent size, encapsulation efficiency, and stability [121]
Nanogels	High loading capacity [122] Stimuli-responsive [123]	Can exhibit certain toxicities depending on their composition [124] Production is often complex and challenging [108]
